# Cooling-induced reactivation of distant faults during long-term geothermal energy production in hot sedimentary aquifers

**DOI:** 10.1038/s41598-022-06067-0

**Published:** 2022-02-08

**Authors:** Iman Rahimzadeh Kivi, Estanislao Pujades, Jonny Rutqvist, Víctor Vilarrasa

**Affiliations:** 1 Institute of Environmental Assessment and Water Research, Spanish National Research Council (IDAEA-CSIC), Barcelona, Spain; 2 Associated Unit: Hydrogeology Group (UPC-CSIC), Barcelona, Spain; 3 Energy Geosciences Division, Lawrence Berkeley National Laboratory, Berkeley, CA USA; 4 Mediterranean Institute for Advanced Studies, Spanish National Research Council (IMEDEA-CSIS), Esporles, Spain

**Keywords:** Hydrogeology, Geophysics

## Abstract

Deep geothermal energy (DGE) represents an opportunity for a sustainable and carbon-free energy supply. One of the main concerns of DGE is induced seismicity that may produce damaging earthquakes, challenging its widespread exploitation. It is widely believed that the seismicity risk can be controlled by using doublet systems circulating water to minimize the injection-induced pressure changes. However, cold water reinjection may also give rise to thermal stresses within and beyond the cooled region, whose potential impacts on fault reactivation are less well understood. Here, we investigate by coupled thermo-hydro-mechanical modeling the processes that may lead to fault reactivation in a hot sedimentary aquifer (HSA) in which water is circulated through a doublet. We show that thermal stresses are transmitted much ahead of the cooled region and are likely to destabilize faults located far away from the doublet. Meanwhile, the fault permeability mainly controls the fault reactivation timing, which entails the importance of employing appropriate characterization methods. This investigation is crucial for understanding the mechanisms controlling induced seismicity associated with DGE in a HSA and allows the success of future DGE projects.

## Introduction

The demand for carbon-free energies is rapidly increasing to face global problems like air pollution and climate change, both resulting from the utilization of fossil fuels. This decarbonization is a challenging task that requires the combination of a suite of technologies. In particular, the energy sector is expected to transform to renewables in the next decades. Most renewable energies suffer from a lack of efficiency since their electricity production is random and intermittent, which makes their utilization difficult^1^. In contrast, geothermal energy has the peculiarity of not fluctuating, guaranteeing the baseload energy demand.

There are different types of geothermal energy that differ on the depth, utilization mode and storage medium^2,3^. Of particular interest is deep geothermal energy (DGE), which refers to systems exploiting the Earth temperature at more than 3000 m and 150 °C^4^. While shallow geothermal energy is mainly used for heating and cooling residential areas, DGE can be used for industrial purposes and to generate electricity^5^. Recent developments with Organic Rankine Cycles (ORC) have permitted to reduce the temperature at which electricity can be generated, extending DGE to shallower depths and temperatures even below 100 °C. DGE can exploit different kinds of geological media, but hot sedimentary aquifers (HSAs) have ideal characteristics for their utilization because of their high geothermal potential^6^.

HSAs have large volumes, high natural porosity and permeability^7^, which means that the geothermal resources (i.e., water) can be used directly. HSAs are usually exploited with a doublet system consisting of a pumping and a reinjection well. The hot water is pumped from the production well, and the waste water (cold water) is returned to the HSA through the reinjection well^5^. The circulating system allows minimizing the surface environmental impact and reduc pressure changes as a result of the geothermal exploitation^8,9^. Reduced pressure changes is particularly important to minimize risks associated with injection-induced seismicity because the injected and produced fluid mass is balanced. Consequently, the pore pressure rapidly reaches a quasi-steady state, which may permit controlling the seismicity risk induced by pore pressure changes. However, low-pressure fluid circulation in sedimentary aquifers has been associated with induced seismic events^10^. Importantly, felt earthquakes of magnitudes M_L_ 2.4 at Unterhaching^11^ and M_L_ 2.1 at Poing^12^ have been recorded after a few months and five years of circulation, respectively, in the Molasse Basin, Germany. The proximity of the earthquakes to Munich has drawn public attention. The temporal and spatial uncertainties of causuality in recorded seismic events necessitate reconsideration of other triggering mechanisms, among them, thermal effects.

Thermal effects may be relevant because the injected fluid is colder than the target formation. The temperature difference arises from the fact that fluid injection through a well becomes isenthalpic after a short transient time and thus, the injected fluid only heats up by compression, yielding a temperature increase with depth much smaller than the geothermal gradient^9^. As a result, a cooled region is formed around the reinjection well that progressively grows with time, advancing mainly by advection. Cooling induces contraction and stress reduction within the cooled region that causes stress redistribution outside it^13–15^. The induced stresses are proportional to the rock stiffness and temperature contrast (see Eq. (1) in “Methods”), so they will be larger in deeper reservoirs of higher temperature and stiffness. Deep formations are also characterized by a higher seismogenic potential, meaning that they are more prone to seismic rupture^16^. The cooled region can reach several hundreds of meters after a few decades of injection^13^, cooling down fractures and faults present in the target formation.

Growing efforts have been dedicated in recent years to a better understanding of thermal effects on induced seismicity. Studies have focused mainly on reservoir stimulation and loss on fluid circulation. The stimulation phase of Enhanced Geothermal Systems (EGS), during which fluid is injected at high pressures to create or reactivate fractures^17,18^, has induced felt damaging earthquakes at Basel^19^, Switzerland, and Pohang^20^, Republic of Korea, causing project cancelations and negatively impacting public perception on geothermal energy. The majority of the conducted studies appreciate cooling effects on induced seismicity limited to the stimulated fractures or fracture zones placed close to the injector^21–27^. The limited spacial scale of thermal effects is due to the smaller characteristic length of the thermal problem compared to the hydraulic problem^22^. These effects may be more pronounced in an extensively fractured reservoir because the higher advection contribution to heat transport promotes the spread of the cooling front^28–30^. Since the cooling front propagation is broadly assessed to be gradual^15,29,31^, cooling-induced fault stability changes may become more important during fluid circulation, rather than during reservoir stimulation. Furthermore, thermoelastic stress penetrates deeper into the reservoir than the cooling front, which may alter the stability of faults placed beyond the cooled region^21^. Accordingly, thermal drawdown may become an important earthquake triggering mechanism in geothermal systems, especially in the long term^15^. This importance is corroborated by observations of induced earthquakes at The Geysers geothermal field, California. The frequency of seismic events of magnitudes *M* > 3 and *M* > 4 increased, respectively, after 20 and 40 years from the start of cold water reinjection^32^, possibly due to cooling-induced stress reduction affecting the stability of faults in the far-field^33^.

**Figure 1:**
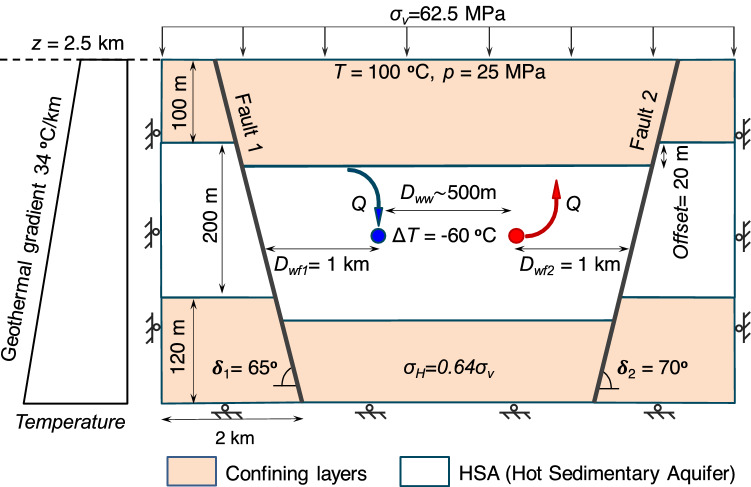
Model setup. The model includes a doublet for geothermal energy production in a Hot Sedimentary Aquifer (HSA) delimited by two normal faults, which may be either of low or high permeability (the sketch is not to scale). The model parameters are summarized in Table 1.

Despite the acquired insights from induced earthquakes in EGS systems, the causal mechanisms of seismicity in HSA on a large scale remains less well understood. The key differences between the two systems originates, on the one hand, from the high-permeability matrix of sedimentary aquifers, promoting flow and thus, the cooling front extension, and on the other hand, from the lower stiffness of sedimentary rock compared to crystalline rock, which lowers the thermal stress reduction induced by a given temperature drop. This investigation aims at advancing the understanding of long-term cooling-induced seismicity in geothermal systems with fluid production and reinjection at a lower temperature. Here, we present the results of coupled thermo-hydro-mechanical (THM) numerical models that simulate a DGE system exploiting with a doublet in a HSA bounded by two normal faults (Fig. 1, see “Methods” for details of the numerical model). We model two scenarios that differ in the permeability of the faults.

## Results

### Geothermal doublet surrounded by low-permeable faults

**Figure 2:**
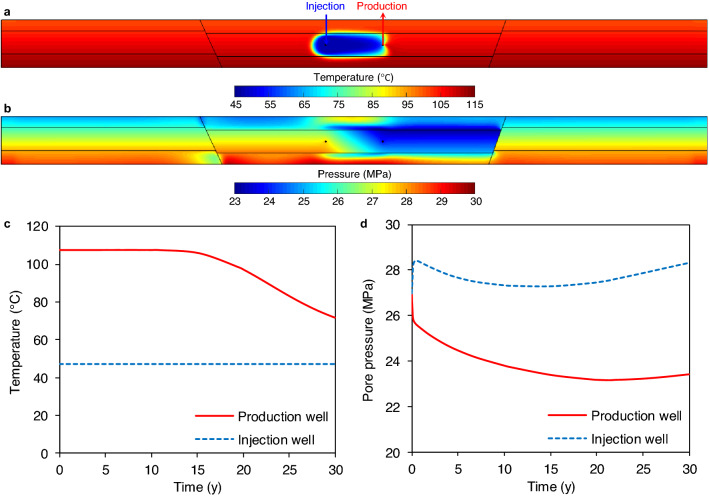
Temperature and pressure evolutions. Spatial distribution of temperature (**a**) and pore pressure (**b**) after 30 years of fluid circulation in a geothermal doublet in a HSA surrounded by low-permeable faults. The evolution with time of temperature (**c**) and pressure (**d**) at the reinjection and production wells.

Cold water reinjection during exploitation of the simulated geothermal reservoir cools down the region around the reinjection well and feeds into heat flow (see Fig. 2a for temperature distribution after 30 years of water circulation, i.e., the end of the simulation). Water temperature ranges from 114 °C (far from the doublet system) to 47 °C (around the reinjection well). Heat transfer across the reservoir is accelerated by advective flow driven by the pore pressure gradient arising between the wells of the doublet (Fig. 2b). The cooling front advances asymmetrically, much faster towards the production well than away from the injector as a result of the larger established hydraulic gradient. The temperature of pumped water starts to decrease after 12 years of utilization (Fig. 2c), known as thermal breakthrough time^34^, and reaches 70 °C after 30 years, progressively degrading the geothermal exploitation efficiency. The slope of the temperature evolution slightly decreases during the last simulated years, a trend expected to continue in the long term. On the left side of the injector, the cooled region is limited to a length of approximately 150 m. The cooling front also approaches the confining layers after 3 years and propagates through them with time mainly by thermal conduction. The cooled-down area in the confining formations after 30 years is vertically confined to half of their thickness and laterally covers the area between the injection and production wells (Fig. 2a).

Pore pressure initially increases around the reinjection well and decreases around the production well, reaching a pseudo-steady state at early times of water circulation that modifies pore pressure within and beyond the HSA (Fig. 2b). Pore pressure across the doublet ranges from 23.4 MPa (production well) to 28.3 MPa (reinjection well) after 30 years of water circulation. Pore pressure variations initially depend on the injection and pumping mass flow rate, which governs the pressure increase and decrease around the reinjection and production wells, respectively (Fig. 2d). Later, pore pressure decreases within the HSA, affecting both wells. This effect is related to the water temperature reduction induced by cold water reinjection that, in turn, increases the fluid density and viscosity. The denser cold water occupies a smaller volume of the pore space compared to the same mass of extracted hot water. As a consequence, pore pressure in the HSA decreases. The trade-off persists between the injection-induced overpressure and cooling effects. As soon as the thermal breakthrough takes place, the density contrast across the doublet begins to diminish while the higher viscosity of the cold water boosts the injection overpressure. As a result, the pore pressure gradually increases at both wells after 15–20 years of water circulation. This increase first appears around the reinjection well from which the cooling effects originate and reaches the production well after some years. Pressure changes within the HSA induce flow across the low-permeable faults and confining layers. Initially, as pore pressure drops within the whole HSA, there is water flow towards the HSA across its boundaries, although at low rates due to their low permeability. Once pore pressure within the HSA increases after 15–20 years of water circulation, the flow direction is reverted, introducing further time-dependency to the problem. Thus, the same trends as in the HSA, but delayed some months or years, dominate the pressure evolution of the confining formations.

**Figure 3:**
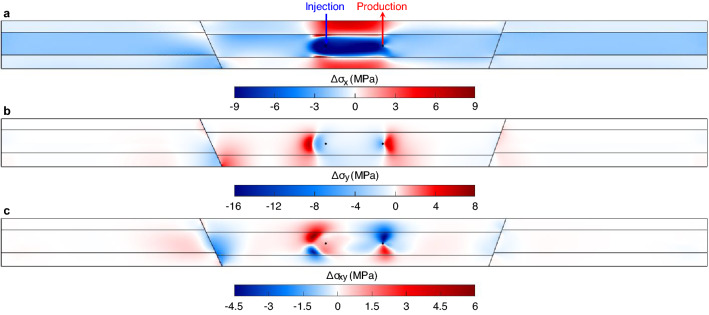
Stress distributions after 30 years of water circulation. Pore pressure changes and thermal effects redistribute the horizontal (**a**), vertical (**b**) and shear (**c**) stresses. Note that the fault slip that has already occurred also contributes to stress redistribution with large vertical stress drop induced along the fault.

Pore pressure and temperature variations give rise to stress changes (see Fig. 3 for stress distribution after 30 years). Pore pressure changes rapidly reach a pseudo-steady state due to the balanced mass circulation within the doublet. In contrast, cooling becomes the dominant process in the long term, contracting the reservoir rock and inducing significant thermal stress reduction inside and outside the cooled region (Fig. 3). The greater the cooling effect, the higher the rock contraction and, thus, the higher the horizontal stress reduction (Fig. 3a). Accordingly, the stress reduction is more intense in the region between the two wells. The displacement continuity and stress equilibrium in the whole system brings the surrounding rock, which is not cooled down and, therefore, does not contract, to forced contraction by generating compressive stresses in the horizontal direction proportional to the rock stiffness^22^. As a result, we can observe positive horizontal stress changes $\Delta \sigma_{x} > 0$ in confining layers and narrow strips, but with higher magnitude, at the top and bottom of the much stiffer reservoir. Regarding the vertical direction, the rock contracts more than in the horizontal direction because the rock is subjected to less deformation constraint vertically due to the free deformation of the ground surface. Therefore, the temperature drop also decreases the vertical stress, but less than in the horizontal direction (Fig. 3b). Analogously to the horizontal stress changes, vertical compressive stress is induced on the lateral boundaries of the cooled region, restraining the rock contraction in the vertical direction. Finally, the non-isotropic decrease of the horizontal and vertical stresses also modifies the shear stress distribution (Fig. 3c). The shear stress is unambiguously concentrated around the cooled region but also in the vicinity of fault 1. The latter is due to fault reactivation after 21 years of water circulation, as will be elaborated in the following sections. One should note that stress variations around the doublet are transmitted across the domain more rapidly than pore pressure perturbations and cooling front advancement^35^. Indeed, the stress tensor is modified in the whole model, implying the potential impacts of thermal stresses on the geomechanical behavior of the geothermal system at distances far away from the doublet.

**Figure 4:**
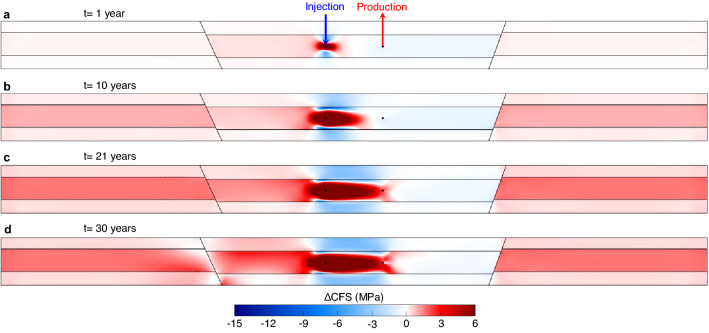
Fault reactivation potential of planes with the same orientation as fault 1. Distribution of the Coulomb Failure Stress changes (ΔCFS) in the direction of fault 1 after 1 (**a**), 10 (**b**), 21 (**c**, corresponding to fault 1 reactivation) and 30 (**d**) years of cold water reinjection. Negative values of ΔCFS imply improved stability while positive values show worsened stability.

The stress evolution caused by cold water reinjection alters the shear and effective normal stresses acting on detected or undetected faults in the system. Therefore, faults may further stabilize or destabilize depending on where they are located and how they are oriented with respect to the principal stresses. Figure 4 shows distributions of the Coulomb Failure Stress changes (ΔCFS, see Eq. (9)) calculated in the direction of fault 1 at several times. The evolution of fault stability conditions tightly correlates with the advance of the cooling front, which can be deduced from comparing Figs. 2a and 4d. The horizontal stress drastically decreases within the cooled region, giving rise to a large reduction of the effective normal stress and increase of the shear stress. Thus, unstable conditions (ΔCFS > 0) occur within this area, indicating that a nearby fault or fracture would be rapidly reactivated and potentially induce (micro)seismicity. On the other hand, the confining layers above and below the cooled region experience an increase in the horizontal stress, which hinders frictional sliding and improves fault stability (ΔCFS < 0). The thermal stresses induce non-negligible shear stress on faults located in the HSA far away from the reinjection well. In addition, pore pressure perturbations advance much ahead of the cooling front and may play a role depending on how they change effective stresses. Stability along these distant faults gradually decreases with time as the cooling front advances. It should be noted that while the pressure drops within the HSA because of the higher density of cold water, fault stability improves. As this stabilizing effect diminishes and the cooling front has advanced significantly, fault 1 eventually reaches failure conditions, which occurs after 21 years of water circulation (Fig. 4c). The slip begins from the upper portion of the fault juxtaposed to the aquifer. The slip induces stress changes within and around the fault. The shear stress releases on the slipping portion, making the fault more stable, while builds up on adjacent fault segments, nucleating the rupture along and beyond the target reservoir (Fig. 4d). In particular, the base rock on the hanging wall of the fault experiences an undrained compression, increasing pore pressure and, thus, the tendency to fail. This observation gives credence to the conjecture that slip along a fault may lead to slip of adjacent faults, or on the contrary, postpone their reactivation^37^. The rupture and the ensuing stress changes last for a few days, highlighting an aseismic slip^36^.

**Figure 5:**
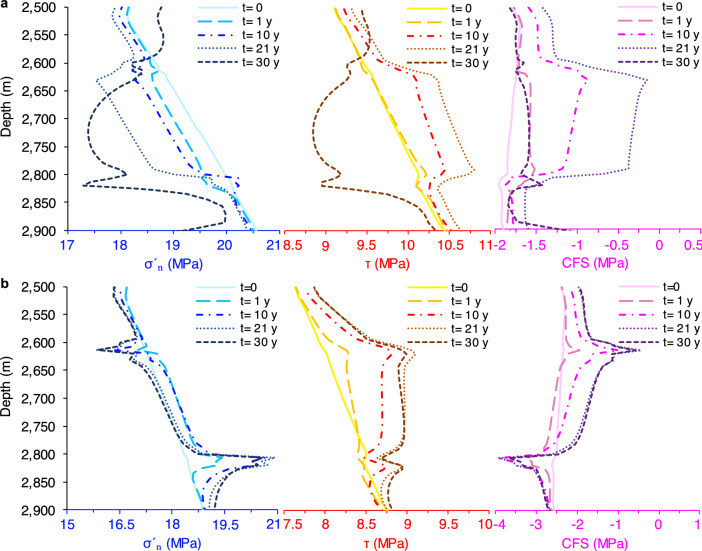
Temporal evolution of fault stability. Evolution with time of the effective normal stress, shear stress and CFS along fault 1 (**a**) and fault 2 (**b**). Colors in each panel gradually shift from light to dark, corresponding to the elapsed time from the circulation onset: Light to dark blue for the effective normal stress, yellow to brown for the shear stress, and light to dark violet for the CFS.

Pore pressure and temperature variations and the resultant stress changes propagate in the reservoir in a thoroughly non-isotropic fashion: the cooling front spreads from the injector while the pore pressure reduction is more pronounced in the region close to the production well. Thus, stability assessment and comparison of the faults located close to each well can be especially insightful with respect to the governing mechanisms. Fault 1 on the left side of the reinjection well experiences slight pressure changes (in the range of 2–3 bars) while stress reductions arise due to cooling effects (see Supplementary Fig. S1 online). Normal stresses decrease along the whole fault plane but more within the reservoir segment because thermal stresses are proportional to the rock stiffness (see Table 1 for material properties). Stress reduction is notably larger in the horizontal direction (for instance, 2 MPa after 21 years in the middle of the HSA compared to a few bars in the vertical direction), which implies enhancement of the deviatoric stress. These stress changes progressively decrease the effective normal stress and increase the shear stress acting on the fault, decreasing fault stability with time until reactivation takes place after 21 years (Fig. 5a). The slip significantly modifies the stress field along and around the fault.

**Table 1:** Material properties of different rock types used in the model.

Parameter	Aquifer	Caprock and base rock	Fault
Rock density, *ρ* (kg/m^3^)	2600	2600	2600
Porosity, $\phi$ (–)	0.2	0.1	0.1
Permeability, $k$ ($m^{2}$)	4 × 10^–14^	1 × 10^–19^	1 × 10^–19^ and 1 × 10^–16^
Thermal conductivity (Wm^−1^ K^−1^)	1.5	1.5	1.5
Specific heat capacity (J kg^−1^ K^−1^)	1000	1000	1000
Thermal expansion coefficient, $\alpha_{T}$ (°C^−1^)	1.5 × 10^–5^	1.5 × 10^–5^	1.5 × 10^–5^
Young’s modulus, $E$ (GPa)	20	5	1
Poisson’s ratio, $\upsilon$ (–)	0.3	0.3	0.35
Biot coefficient, $\alpha$ (–)	1	1	1
Cohesion, c (MPa)	–	–	0
Initial friction angle, $\varphi^{ini}$ (°)	–	–	31
Residual friction angle, $\varphi^{res}$ (°)	–	–	29
Dilation angle, $\psi$ (°)	–	–	4
Softening parameter, $\eta^{*}$ (–)	–	–	0.005
Viscosity, $\mu_{vp}$ (MPa^m^s)	–	–	10^4^

The behaviour of fault 2 differs from that of fault 1 in that pore pressure decreases with time due to the fault proximity to the production well. The pressure drop is larger, representing a maximum value, in the lower part of the fault, where it is juxtaposed with the base of the HSA on the hanging wall and the base rock on the footwall (see Supplementary Fig. S2 online). As an example, after 21 years of circulation, the pressure drop in this fault portion becomes as high as $\approx -$ 3.6 MPa, which is twice that induced in the middle of the fault. This anomaly forms because the low-permeability base rock acts as a flow barrier to lateral flow, which tends to balance pressure changes. The pore pressure decrease causes the rock to compress, which superposes cooling effects in decreasing normal stress. The stress reduction is larger in the horizontal direction but progressively becomes smaller towards the abovementioned lower fault segment. This stress decay around the lower part is due to the fact that the base rock is four times softer than the reservoir rock, undergoing more deformation and, therefore, allowing for less stress accumulation. The induced deviatoric stress gives rise to shear stress build-up through the whole fault. Contrarily, the effective normal stress only decreases in the upper portion and increases in the rest of the fault (Fig. 5b). In a nutshell, while thermal stresses promote shear failure of fault 2, the pore pressure drop delays it. The superposition of these processes improves fault stability in the segment adjacent to the base rock while promoting fault reactivation in the overlying part. Nevertheless, fault 2 does not reactivate during the water circulation period because the slip along fault 1 prevents further shear stress build-up along the upper portion of fault 2, in spite of the long distance.

### Effect of fault permeability

**Figure 6:**
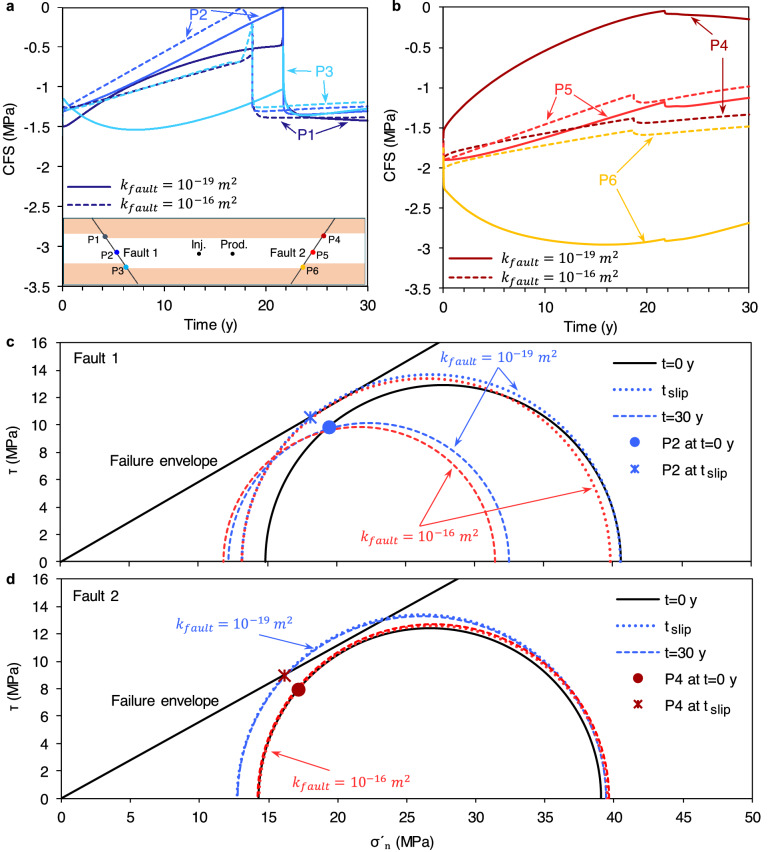
Impact of fault permeability on its stability. Evolution with time of the slip tendency (CFS) at the top, middle and bottom of fault 1 (**a**, corresponding to dark to light blue, respectively) and fault 2 (**b**, corresponding to brown, red and orange, respectively). The most critical stability conditions are reached in the middle of fault 1 (point 2) and at the top of fault 2 (point 4). (**c**,**d**) Mohr circles are drawn at these two points before circulation (t = 0), and after t_slip_ ($\approx$ 18 and 21 years for high- and low- permeability faults, respectively) and t = 30 years of water circulation. One should note that minor stress changes on fault 2 occur after the slip; thus, Mohr circles of *t*_slip_ and *t* = 30 years almost coincide.

The presence of relatively high-permeability faults allows for effective fluid communication of the HSA with the surrounding reservoir rock on the other side of the faults. Therefore, the geomechanical behavior of the geothermal system differs from that explained for compartmentalized reservoirs. We compare the stability of low-permeability (*k* = 10^–19^ m^2^) and high-permeability (*k* = 10^–16^ m^2^) faults in terms of the CFS evolution with time at three points at the top, middle and bottom of the HSA (Fig. 6). For the high-permeability faults, the pressurized region around the reinjection well rapidly extends laterally and continuously builds up pressure on fault 1. The size of the pressurized fault patch progressively increases with time as water diffuses upward and downward along the fault. The overpressure causes a reduction of the effective normal stress, which superposes to the cooling-induced stress reduction taking place mostly in the horizontal direction. The resulting effective stress changes enlarge the Mohr circle and simultaneously shift it towards the failure envelope, thus driving the fault to unstable conditions (Fig. 6c). The slip arguably begins aseismically in the middle of the aquifer after $\approx$ 18 years and then converts into a growing aseismic rupture as the stress redistribution causes instability along a large fault patch (Fig. 6a). This behavior differs from that of the low-permeability fault, where thermally-induced deviatoric stress dominates fault stability. Furthermore, the increased permeability advances the fault slip in $\approx$ 3 years (compared to *t*_failure_ = 21 years for the low-permeability fault) because the initial pressure drop in the case of the compartmentalized reservoir disappears. Such a transient stability variation is featured at the lower segment of the fault adjacent to the base rock, where the fault stability (dashed line for P3 in Fig. 6a) and the reservoir pressure (Fig. 2d) changes follow almost the same trend. The observed differences in the reservoir pressure evolution can also corroborate the stability contrasts emerged on fault 2 (Fig. 6b). The pore pressure continuously decreases on the high-permeability fault in the proximity of the production well. The effective normal stress increases with time, moving the Mohr circle away from the failure envelope and partially counterbalancing thermally-driven shear stresses, which is responsible for the Mohr circle growth (Fig. 6d). As a result, while the low-permeability fault 2 locally approaches failure conditions (point P4) and, concomitantly, notably stabilizes on the lower portion (point P6), the high-permeability fault 2 witnesses slight and practically uniform stability reduction during the 30 years of water circulation (Fig. 6d). Surprisingly, reactivation of fault 2 is hampered by slip along fault 1. The shear stress drop resulting from the slip of fault 1 (Fig. 6c) overall delays the elastic energy build-up on fault 2, reducing its slipping tendency. As a result, one can observe that the Mohr circles of fault 2 at the slip time and after 30 years almost coincide.

## Discussion

One of the main concerns of deep geothermal exploitation is induced seismicity. Two concomitant mechanisms during water circulation are found to control the mechanical behavior of geothermal systems: pore pressure diffusion and thermal effects. The former, which correlates with the established pore pressure gradient between the doublet, plays a first-order role in the short term (in the order of some days to months) because the fluid front travels through the reservoir rock matrix orders of magnitude faster than the cooling front. The pore pressure increase may destabilize faults by reducing the effective normal stress acting on the fault plane, i.e., bringing the Mohr circle towards the failure envelope. However, this process is, in practice, of minor significance due to the balanced injection/production mass flow rate that prevents excessive pore pressure build-up. Developing a doublet system in a compartmentalized reservoir is found to further smooth pore pressure changes as the injected cold water is denser than the extracted hot water, yielding a lower injection than production rate and occupying less space in the pore system. On the other hand, thermal effects are quite appreciable in the long term (in the order of years to decades). Cooling-induced horizontal stress reduction increases the deviatoric stress on the fault, enlarging the Mohr circle and shifting the center of the Mohr circle towards the failure envelope, which leads to unstable conditions. Interestingly, an initially not critically oriented fault, located almost 1 km away from the reinjection wellbore, reactivates even though the cooling front does not propagate more than 150 m laterally in the reservoir (Fig. 2a). Indeed, the local thermal stresses around the doublet build up on the acting background stress state to affect a wider region on a field-scale basis. Simulation results highlight the potential of cold water reinjection to reactivate faults at long distances from the cooled region. Consequently, even if the fluid mass injection and withdrawal rates are balanced in geothermal systems, the likelihood of inducing earthquakes increases as the cooled region grows.

The slip is primarily initiated by thermal stresses, but after the onset of slip, the static stress transfer jointly contributes to the rupture nucleation along the fault. The slip gradually releases the stored elastic energy over a few days, as could be expected from the small drop in friction angle from $\varphi = 31$° to $\varphi = 29$°. This slip-weakening behavior is consistent with laboratory and field observations that a large part of the slip in the upper-crust zone is aseismic^38–40^. However, the aseismic slip may build up stress on the underlying layers, where the crust is more seismogenic, e.g., the crystalline basement, augmenting the likelihood of large seismic events. Such stress transfer is perceived to be the triggering mechanism of the felt earthquakes that occurred several kilometers below the Brawley geothermal field^41,42^, California, and the Castor gas storage site^43^, Spain. Conversely, our observations support the view that the destressing driven by aseismic slip can impede or delay an earthquake in nearby faults (Fig. 6b)^44^.

The permeability of faults plays a key role: the more permeable the fault is, the earlier it reactivates. In the high-permeability fault scenario, the fault begins to reactivate roughly after 18 years when the thermal breakthrough of the doublet has not practically occurred and the geothermal system is economically worth continuing operation. The hydraulic properties of the exploited reservoir, the injection/production mass flow rate, the injection temperature, and the fluid properties control the established hydraulic and thermal gradient, and thus, the needed time to reactivate faults by cooling-induced effects. In this sense, if CO_2_ is used as the working fluid, owing to its higher heat extraction capacity and low viscosity compared to water, geothermal energy production should be more efficient and, concurrently, CO_2_ emissions are reduced^45^. Nevertheless, CO_2_ density drastically changes due to temperature variations when flowing across the reservoir. For instance, it decreases from 870 kg/m^3^ down to 606.3 kg/m^3^ when the temperature evolves from 47 to 107 °C at a pressure of 28 MPa^46^. The large density difference between the injected cold CO_2_ and produced hot CO_2_ may impose pressure management challenges in compartmentalized reservoirs. Two-phase flow numerical models should therefore be adopted to investigate how cooling-induced stress reductions may affect the stability of faults placed within and far away from the cooled region when using CO_2_ as working fluid. Furthermore, the influence of the reservoir characteristics and operational parameters on induced seismicity in hot sedimentary formations should be recognized and ranked. Indeed, our observations are founded upon arbitrary rock properties. The THM properties of the aquifer and confining layers are chosen as those of well-characterized Berea sandstone and Opalinus Clay^47^, respectively, which represents a typical HSA behavior^48,49^. However, the aquifer may possess higher thermal expansion coefficient and stiffness^50,51^, promoting thermoelastic stresses and, possibly, accelerating fault reactivation (see Eq. (1)). On the other hand, in spite of assuming a Biot coefficient equal to 1, which intensifies poroelastic stress perturbations, poroelastic controls on fault stability are negligible compared to direct pore pressure diffusion and, thus, are not discussed in this paper. Finally, although the expansion coefficient of the caprock can be twice as large as the set value^52^, it is unlikely to affect the flow and deformation behavior of the aquifer and distant faults as the cooled region within the caprock is constrained to a narrow strip at its bottom.

While major seismic events are expected to stem from slip along the main fault(s), probably due to large rupture area, thermal contraction can also bring the fractured rock to failure and produce microseismicity^28–30^. Similar clouds of small-magnitude earthquakes (*M* ≤ 3) have been observed to temporally correlate well with cold water reinjection trends and progressively extend away from the injectors in The Geysers geothermal field^32,53,54^. The seismic cloud has moved downwards on the order of 100 m per year due to progressive cooling as the denser cold water moved downwards by gravity^54,55^. Moreover, a seismically quiet zone developed next to the reinjection well was explained by cooling, while seismicity progressed outside this quiet zone^56^. Cooling through the permeable fault network leads to complex THM responses where the largest magnitude events, i.e., *M* > 3, may typically occur at the periphery or seismic front, in particular near bounding faults^57^. Such instabilities may dilate the fractured rock and fault and enhance their permeabilities, which, in turn, give rise to pore pressure and stress redistribution with non-trivial impacts on the geomechanical behavior of the system. The effect of stress-dependent permeability is more pronounced in low-permeability structures, especially in confining layers and tight fault segments, where undrained deformation prevails^47^. In such circumstances, assuming the cubic relationship between transmissivity and aperture of fractures^58^, small increments of fracture aperture are associated with enhancing the equivalent rock permeability by orders of magnitude, extending the pressurized area.

Despite the spatial extent within which faults may be affected and the timing and magnitude of potential earthquakes are site-specific, the coupled processes highlighted here that govern the geomechanical response of the HSA system are universal. Numerical modeling should be employed to identify the optimum injection conditions for each individual geothermal site subjected to well-characterized geological structures, reservoir rock, and fault physical properties. In particular, pre-existing faults should be identified both within and around the target reservoir affected by cooling-induced stress changes that extend far away from the cooled region. Conclusively, a deep geological characterization is an essential part of best field practices for appropriate reservoir management that avoids fault reactivation to sustain the production of geothermal resources over a long period.

## Methods

### Numerical model

We consider an ideal horizontal, homogeneous and isotropic HSA bounded by opposite dipping normal faults with dip angles of 65° (fault 1) and 70° (fault 2) and an offset of 20 m (Fig. 1). The model is extended laterally 2 km beyond the two faults to minimize boundary effects on simulation results. The aquifer is underlain and overlain by low-permeability base rock and caprock, respectively (see Table 1 for rock properties). We assume plane strain conditions, which can be justified regarding the problem geometry, including two horizontal wellbores extending in the out-of-plane direction. The top of the 200-m-thick HSA is located at a depth of 2500 m. The initial pressure distribution is hydrostatic. The initial temperature is 100 °C at the top of the model, increasing with depth with a gradient of 34 °C/km. The in situ stress reproduces a normal faulting stress regime, with the overburden stress corresponding to the maximum principal stress and varying with a 25 MPa/km gradient. The horizontal stress is isotropic and equals 0.64 times the vertical stress. We conduct a steady-state calculation to bring pressure, temperature and stress fields to an initial equilibrium.

Hot water is pumped from a production well, placed at a depth of 2720 m and 1 km away from fault 2, and cold water is reinjected through the injector, placed at the same depth as the injector, and 1 km away from fault 1. The water mass flow rate is the same in both wells and is maintained constant for 30 years. The temperature of the injected water is constant (47 °C), causing a temperature difference of 60 °C with the reservoir, while that of the production well starts from 107 °C and varies as the cooling front advances. The hydraulic boundary conditions are hydrostatic pressure on lateral boundaries and no flow on the top and bottom boundaries. We impose a constant temperature profile (with a gradient of 34 °C/km) on lateral boundaries and no thermal flow at the top and bottom of the model. The mechanical boundary conditions are constant overburden stress of 62.5 MPa at the top and zero displacements normal to other boundaries. We numerically solve the explained themo-hydro-mechanical problem using the fully-coupled finite element code CODE_BRIGHT^59^.

### Model constitutive equations

The relationship between stresses, strain, fluid pressure and temperature are given by Hooke’s law,

1 $$\Delta {\varvec{\sigma}} = K\varepsilon_{v} {\varvec{I}} + 2G\left( {{\varvec{\varepsilon}} - \frac{{\varepsilon_{v} }}{3}{\varvec{I}} + \frac{\alpha }{2G}\Delta p_{f} {\varvec{I}} - \frac{3K}{{2G}}\alpha_{T} \Delta T{\varvec{I}}} \right)$$

where ${\varvec{\sigma}}$ is the total stress tensor, $\varepsilon_{v}$ is volumetric strain, ${\varvec{I}}$ is the identity matrix, ***ε*** is the strain tensor, $K = E/(3(1 - 2\nu ))$ is the bulk modulus, $G = E/(2(1 + \nu ))$ is the shear modulus, *E* is Young’s modulus, $\nu$ Poisson’s ratio, $p_{f}$ is the fluid pressure, $\alpha$ is the Biot effective stress coefficient, $\alpha_{T}$ is the linear thermal expansion coefficient and *T* is temperature. Notice that stresses vary with temperature according to $3K\alpha_{T} \Delta T{\varvec{I}}$, which means that variations of temperature produce large variations of stresses in stiff rocks.

The mechanical problem is solved satisfying the momentum balance. If the inertial terms are neglected, supporting a quasi-static deformation, it reduces to the equilibrium of stresses

2 $$\nabla \cdot {\varvec{\sigma}} + {\varvec{b}} = 0$$

where **b** is the vector of body forces.

Equation (1) can be coupled with the flow equation considering the fluid pressure. Assuming that there is no external loading and neglecting solid phase compressibility, fluid mass conservation of the fluid can be written as

3 $$\left( {\frac{\Phi }{{K_{f} }} + \frac{1}{K}} \right)\frac{{\partial p_{f} }}{\partial t} + \frac{d}{dt}\left( {\nabla \cdot {\varvec{u}}} \right) + \nabla \cdot {\varvec{q}} = 0,$$

where $\Phi$ is porosity, $1/K_{f}$ is water compressibility, *t* is time, **u** is the displacement vector and ${\varvec{q}}$ is the water flux, given by Darcy’s law. Notice that the flow (Eq. (3)) and mechanical (Eq. (1)) equations can also be coupled through the volumetric strain (second term in the left-hand side of Eq. (3)), which can be expressed as the divergence of the displacement vector.

### Fault constitutive equations

Here, we provide a brief explanation of the viscoplastic constitutive model used to simulate the fault behavior. The model is written in terms of three main functions as follows

4 $$F = P\cdot\sin \varphi (\eta ) + (\cos \theta \cdot\sin \varphi (\eta )) \cdot \sqrt {J_{2} } - c(\eta ) \cdot \cos \varphi (\eta )$$

5 $$g = \beta \cdot P \cdot \sin \psi + \left( {\cos \theta - \frac{1}{\sqrt 3 }\sin \theta \cdot \sin \psi } \right) \cdot \sqrt {J_{2} } - c(\eta ) \cdot \cos \varphi (\eta )$$

6 $$\left\langle {\Phi \left( F \right)} \right\rangle = \left\{ {\begin{array}{*{20}c} {0,} & {\quad if\; F \le 0} \\ {F^{m} ,} & {\quad if\; F > 0} \\ \end{array} } \right.$$

where $F$ is the yield function, corresponding to the Coulomb criterion, $g$ is a non-associated viscoplastic potential function, $P$ is the effective mean stress, $J_{2}$ is the second invariant of the deviatoric stress tensor, $\theta$ is Lode’s angle, c is cohesion, $\varphi$ is friction angle, $\eta$ is the softening parameter (or plastic shear strain), $\beta$ is a non-associativity parameter, $\psi$ is dilatancy angle, $\Phi (F)$ is the overstress function, which defines the viscoplastic limit, and $m$ is a constant power, chosen equal to 3 here. The initial state of stress entails $F < 0$, which means the fault deforms in an elastic or viscoelastic manner. Once the stresses approach the shear yield surface ($F > 0$), the fault begins to slip. The magnitude of the viscoplastic deformation is determined using the flow rule, which writes as

7 $$\frac{{d\varepsilon_{vp} }}{dt} = \frac{1}{{\mu_{vp} }}\left\langle {\Phi \left( F \right)} \right\rangle \frac{\partial g}{{\partial \sigma }}$$

where $\varepsilon_{vp}$ and $\mu_{vp}$ are viscoplastic strain and viscosity, respectively. In contrast to purely plastic theories, the viscoplastic model allows the stress to exceed the yield surface and reside in a range determined by the overstress function^60^.

The assessment of the onset of fault slip is carried out using the static friction coefficient. The slip progressively deteriorates the previously interlocked asperities along the fracture surfaces. As a result, the fault strength properties degrade with the plastic shear strain. Accordingly, the fault strength is hypothesized to weaken linearly from the peak static value $\varphi^{ini}$ to a residual dynamic value $\varphi^{res}$ over a critical plastic shear strain $\eta^{*}$, as follows

8 $$\varphi (\eta ) = \left\{ {\begin{array}{*{20}l} {\varphi^{ini} } & {\quad \eta \le 0} \\ {\varphi^{ini} + \left( {\frac{{\varphi^{res} - \varphi^{ini} }}{{\eta^{*} }}} \right) \cdot \eta } & {\quad 0 \le \eta \le \eta^{*} } \\ {\varphi^{res} } & {\quad \eta^{*} \le \eta } \\ \end{array} } \right.$$

If the friction coefficient drops rapidly, seismic events are expected, while a gradual loss of friction corresponds to aseismic slip^36^. Unambiguously, the fault begins to slip along the most critically stressed segment (finite element mesh). The friction weakening behavior features a stress drop proportional to the difference between static and dynamic friction coefficients. Stress transfers to neighboring fault segments, potentially promoting conditions for slip along them and, thus, mimicking progressive fault rupture. The explained constitutive model has been found to be computationally efficient and yet realistic in capturing the physics of earthquake nucleation and propagation^61,62^.

### Stability assessment in a particular direction

We use the Coulomb Failure Stress (CFS) to track the slip potential in any direction of interest across the model

9 $$CFS = \left| \tau \right| + {\sigma^{\prime}}_{n} \tan (\varphi^{ini} )$$

where $\tau$ and ${\sigma^{\prime}}_{n}$ are shear stress and effective normal stress acting on a plane, respectively. The stresses and, thus, the CFS depend on the dip direction and angle of the plane. Numerical simulations show that slip occurs only along fault 1. Therefore, we consider the direction of fault 1 for CFS evaluation throughout the paper. Furthermore, variations of the CFS with respect to its initial magnitude (ΔCFS) indicate evolutions of the fault stability with time. Accordingly, ΔCFS > 0 shows decreased stability along possible fractures and faults in the studied area with the same direction as fault 1.

## Supplementary Information

Supplementary Figures.

## Data Availability

The input files of the numerical models are available at the institutional repository Digital.CSIC (http://hdl.handle.net/10261/252456). All numerical simulations, the presented results and findings of this study can be reproduced by the provided data.

## Code availability

The numerical code used in this study is open source and can be downloaded at https://deca.upc.edu/en/projects/code_bright/downloads.
