# Supplementary material for: Cooling-induced reactivation of distant faults during long-term geothermal energy production in hot sedimentary aquifers
Source: Sci Rep. 2022 Feb 8;12:2065. doi: 10.1038/s41598-022-06067-0 (PMC8826403; doi:10.1038/s41598-022-06067-0)
Supplement: Supplementary file 1 — Supplementary Figures. [file 41598_2022_6067_MOESM1_ESM.pdf]

Supporting Information for

Cooling-induced reactivation of distant faults during long-term geothermal energy production in hot sedimentary aquifers

Iman Rahimzadeh Kivi[1,2]*, Estanislao Pujades[1,2], Jonny Rutqvist[3] and Víctor Vilarrasa[1,2,4]

[1] Institute of Environmental Assessment and Water Research, Spanish National Research Council (IDAEA-CSIC), Barcelona, Spain

[2] Associated Unit: Hydrogeology Group (UPC-CSIC), Barcelona, Spain

[3] Energy Geosciences Division, Lawrence Berkeley National Laboratory, CA, USA

[4] Mediterranean Institute for Advanced Studies (IMEDEA), Spanish National Research Council (CSIC), Esporles, Spain

email: iman.rahimzadeh@idaea.csic.es*

Introduction

We present in the Supporting Information the evolution of the pore pressure and stress components along faults 1 (Fig. S1) and 2 (Fig. S2), for the case in which both faults have a low permeability.

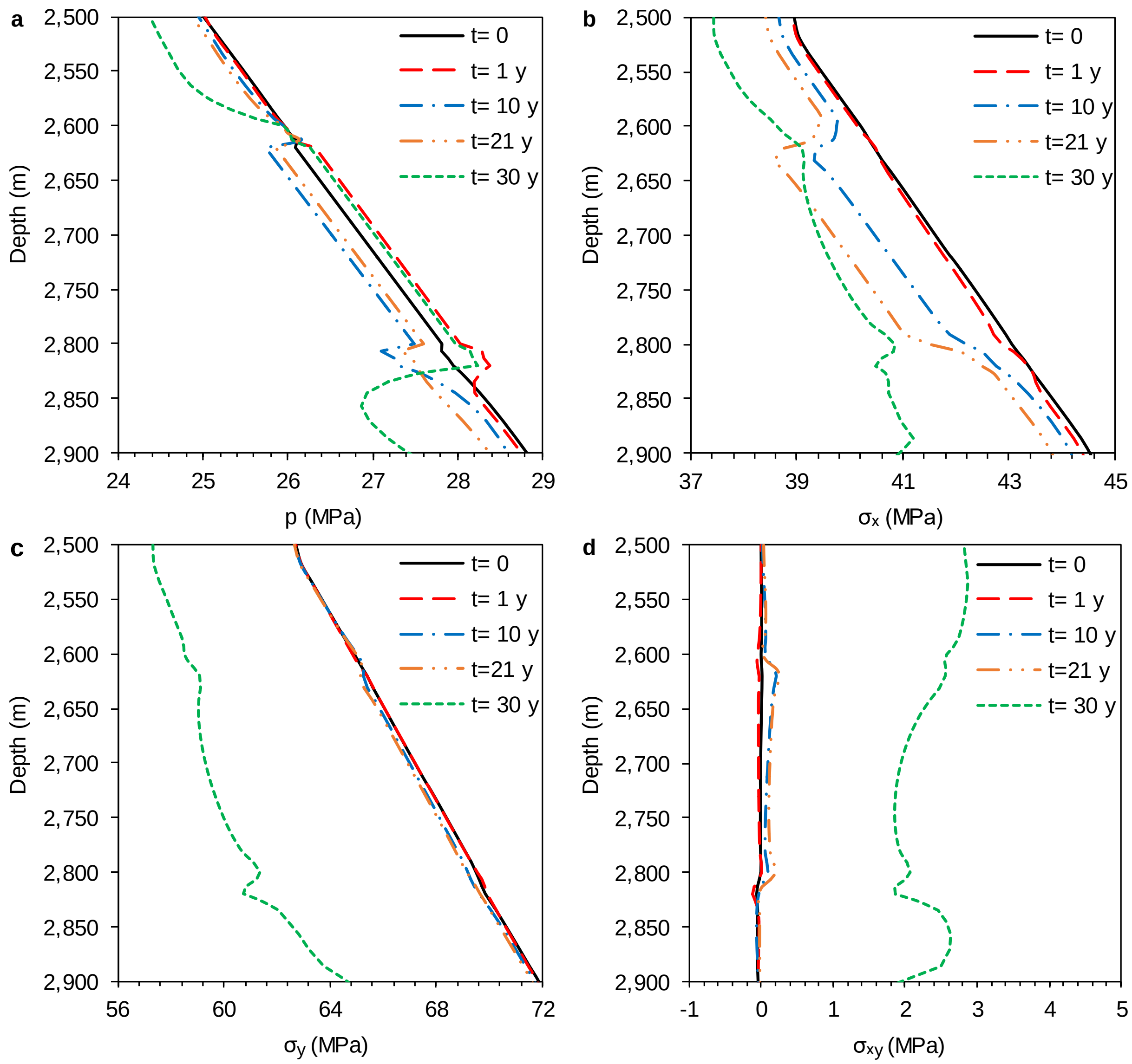

Figure S1. Pore pressure and stress evolution in fault 1. Evolution of the pore pressure (**a**), horizontal stress (**b**), vertical stress (**c**) and shear stress (**d**) along fault 1 at different times during cold water reinjection.

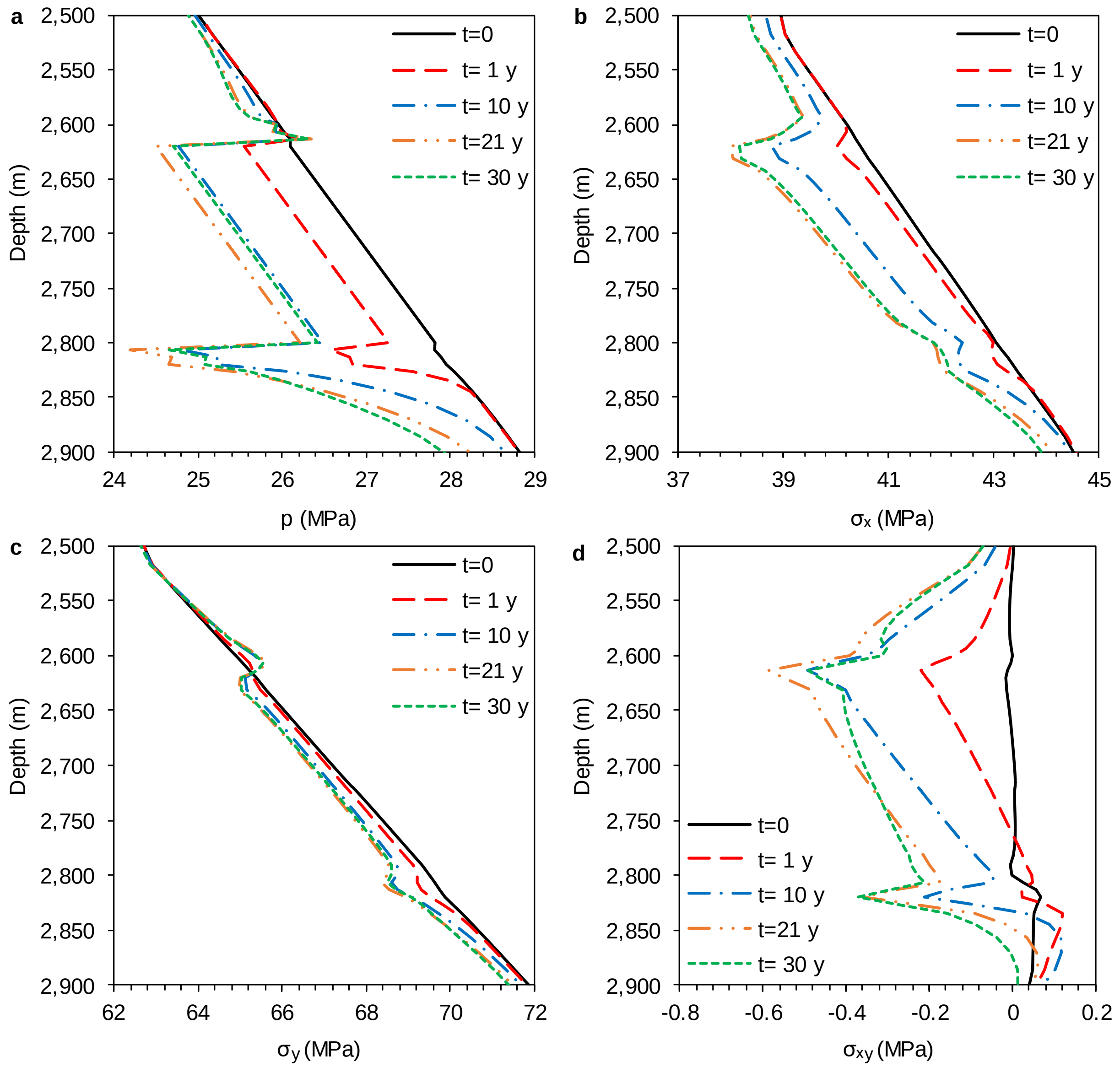

Figure S2. Pore pressure and stress evolution in fault 2. Evolution of the pore pressure (**a**), horizontal stress (**b**), vertical stress (**c**) and shear stress (**d**) along fault 2 at different times during cold water reinjection.
